# Predictors of willingness to patronize traditional bone setters: a cross-sectional study among heads of households in Abakaliki Metropolis, Southeast Nigeria

**DOI:** 10.11604/pamj.2024.49.106.36444

**Published:** 2024-12-03

**Authors:** Edmund Ndudi Ossai, Ifeyinwa Lilian Ezenwosu, Kelechukwu Anthony Okoro, Irene Ifeyinwa Eze, Chibuike Agu

**Affiliations:** 1 Department of Community Medicine, Alex Ekwueme Federal University Teaching Hospital, Abakaliki, Nigeria,; 2 Department of Community Medicine, University of Nigeria Teaching Hospital Ituku-Ozalla, Enugu, Nigeria,; 3 Department of Surgery, University of Nigeria Teaching Hospital Ituku-Ozalla, Enugu, Nigeria

**Keywords:** Willingness, traditional bone setters, heads of household, Abakaliki Metropolis, Southeast Nigeria

## Abstract

**Introduction:**

the traditional bone setters provide services that are commonly associated with complications that could be life-threatening. Nevertheless, these do not affect the proportion of people who patronize traditional bonesetters. This study aimed to determine the predictors of willingness to patronize traditional bone setters among heads of households in Abakaliki Metropolis, Southeast Nigeria.

**Methods:**

this was a community-based cross-sectional study. A four-stage sampling design was used to select 420 heads of households from Abakaliki Metropolis, Nigeria. Information was obtained using a pre-tested, interviewer-administered questionnaire which was developed by the researchers. The study employed Chi-square test and multivariate analysis using binary logistic regression.

**Results:**

less than a third of the respondents, 32.4% were willing to patronize traditional bone setters in the future and the major reasons included reduced treatment cost, 45.6% and good treatment services, 29.4%. Predictors of the willingness of heads of household to patronize traditional bone setters in the future included being aged 30-39 years, (AOR=0.5, 95%CI: 0.2-0.9), being in low socio-economic class, (AOR=2.0, 95%CI: 1.2-3.3), having patronized traditional bone setters before, (AOR=12.5, 95%CI: 7.5-20.8) and being in support of surgeons and bone setters working together, (AOR=2.7, 95%CI: 1.6-4.4).

**Conclusion:**

cost and good treatment services play roles in the patronage of bone setters. An appreciable number of respondents are willing to patronize them in the future. There is the need for bone setters to concentrate on areas of competence bearing in mind that there are measures of good in their services. They should be trained on good referral practices.

## Introduction

The traditional bone setting is a specialization in traditional medicine that entails informal training among family members as part of ancestral heritage or to non-family members as an apprenticeship [1]. Traditional bone setters are unqualified practitioners who take up the practice of joint and bone manipulations without any formal training in modern medical procedures [2]. Though modern healthcare services have markedly improved due to advancements in technology and medical research, the traditional bone setting is still practiced as an alternative healthcare service [3].

Globally, an estimated 10-40% of people with dislocations and fractures are managed by traditional bonesetters [4]. In recent decades, there has been an increase in the number of road traffic accidents and the burden of musculoskeletal injury in sub-Saharan Africa, resulting in a higher demand for musculoskeletal care [5]. However, due to limited access to orthopaedic care, many people in African countries sought care from unorthodox practices [5]. The traditional bone setters render services that are commonly associated with complications that could be life-threatening such as tetanus, gangrene, and sepsis [6]. The complications are linked to the lack of basic scientific knowledge in human anatomy, physiology, radiology, and basic principles of infection prevention in the practices of traditional bonesetters [2,6]. Up to 85% of those with fractures and bone injuries in Nigeria are treated by traditional bonesetters before presenting to orthopaedic facilities after developing gangrene [7].

Nevertheless, these do not affect the proportion of people who patronize traditional bonesetters [6]. There are various reasons why people patronize unorthodox practices, evidence has shown that socio-cultural beliefs, ignorance, easy accessibility, relatively cheaper cost of treatment, perceived quicker services, and pressure from relatives and friends are the basis for continued patronage [2]. Furthermore, individuals from different socio-economic classes, religious beliefs, occupations, and levels of education patronize traditional bonesetters for their services [8].

It is believed that heads of households can influence the decisions of their family members regarding the utilization of health care [9]. Furthermore, most members of a household tend to follow their various heads in patronizing health services as approved by the heads including unorthodox practices [10]. Though several studies have been done on the willingness to patronize traditional bone setters in different categories of people, very few studies have accessed the heads of households, who are decision-makers in the family and could influence the type of health care services their family members will utilize [2,6]. Therefore, this study accessed the willingness to patronize traditional bone setters and its predictors among heads of households in Abakaliki Metropolis, Southeast Nigeria.

## Methods

**Study setting:** Abakaliki is the administrative capital of Ebonyi State, Southeast Nigeria. The metropolis consists of three local government areas (LGAs) including Abakaliki, Ebonyi, and Izzi LGAs. It is also made up of seven districts. The inhabitants, like those of other states in southeast Nigeria, are predominantly of Igbo ethnicity. The metropolis has the highest concentration of health facilities in the state including two tertiary health institutions.

**Study design:** the study adopted a community-based cross-sectional study design.

**Study population:** the heads of households in the metropolis constituted the study population. A household is a group of individuals, biologically related or not, living together and eating from a common kitchen [11]. The head of a household is the individual in the household who is responsible for leadership and financial decisions in the household [11].

**Sample size determination and sampling technique:** this has been described in a previous publication [12].

**Study instrument:** a pre-tested, semi-structured questionnaire was used for data collection. The questionnaire was designed by the researchers and was administered to the respondents by trained research assistants. The questionnaire was pre-tested in another LGA in the state outside the metropolis.

**Data management:** data entry and analysis were done using IBM Statistical Package for Social Sciences (SPSS) version 25. Categorical variables were summarized using frequencies and proportions while continuous variables were presented using mean and standard deviation. Chi-square test and multivariate analysis using binary logistic regression analysis were used in the study and the level of statistical significance was determined by a p-value of <0.05.

The outcome measure of the study was the willingness of heads of households to patronize traditional bone setters in the future. This was determined by the response to one variable; Are you willing to patronize traditional bone setters in the future? In determining the factors that affect the willingness of heads of households to patronize traditional bone setters in the future, variables that had a p-value of <0.2 on bivariate analysis were entered into the logistic regression model to determine the predictors of willingness to patronize traditional bone setters in the future. The result of the logistic regression analysis was reported using an adjusted odds ratio and 95% confidence interval. The level of statistical significance was determined by a p-value of <0.05. The determination of the socio-economic class of the heads of households has been described in a previous publication [12].

**Ethics approval and consent to participate:** ethical considerations: the Research and Ethics Committee of Ebonyi State University Abakaliki, Nigeria gave ethical approval for the study. The reference number is EBSU/DRIC/UREC/vol. 04/064. Before participating in the study, the respondents were required to sign a written informed consent form, and the nature of the study as well as the level of their participation were explained to them. Confidentiality of their information was assured.

## Results

The socio-demographic characteristics of the respondents have been described in a previous publication [12].

**Willingness to patronize traditional bone setters among heads of households:** Table 1 shows that more than three-quarters (79.3%) of the respondents have heard of traditional bone setters and 32.4% of them will patronize them in the future. The major reasons for willingness to patronize them in the future were reduced cost of treatment (45.6%) and good treatment services (29.4%). Though the majority of the respondents (43.3%) reported that traditional bone setters have more treatment failures compared to orthopedic surgeons, 33.1% of them will encourage their family members to patronize them and 33.9% of the respondents support surgeons and bone setters working together in the same setting.

**Table 1:** willingness to patronize traditional bone setters among heads of households

Variable	Frequency (n=420)	Percent (%)
**Have heard of traditional bone setters**		
Yes	333	79.3
No	87	20.7
**Have patronized traditional bone setters before**		
Yes	140	33.3
No	280	66.7
**Will patronize bone setters in the future**		
Yes	136	32.4
No	170	40.5
Don’t know	114	27.1
**Reason to patronize traditional bone setters**	**(n=136)**	
Reduced cost of treatment	62	45.6
Good treatment services	40	29.4
Avoid amputation	16	11.8
Not certain	18	13.2
**Encourage family members to patronize bone setters in the future**	**(n=420)**	
Yes	139	33.1
No	164	39.0
Uncertain	117	27.9
**The group that has more patronage between surgeons and bone setters**		
Orthopedic Surgeons	184	43.8
Traditional bone setters	162	38.6
Uncertain	74	17.6
**The group personally preferred treatment**		
Orthopedic surgeons	243	57.9
Traditional bone setters	115	27.4
Uncertain	62	14.8
**The group that has more treatment failures**		
Traditional bone setters	182	43.3
Orthopedic surgeons	103	24.5
Uncertain	135	32.1
**The group that offers more expensive treatment**		
Orthopedic surgeons	359	85.5
Traditional bone setters	16	3.8
Uncertain	45	10.7
**Supports surgeons and bone setters working together in the same setting**		
Yes	165	39.3
No	152	36.2
Don’t know	103	24.5

**Reasons why people patronize traditional bone setters:** the respondents noted that the major reasons people patronize traditional bone setters were affordability of treatment (51.0%), followed by good treatment experience (17.1%) and ignorance (10.7%) (Table 2).

**Table 2:** reasons why people patronize traditional bone setters

The reason why people patronize traditional bone setters	Frequency (n=420)	Percent (%)
Cheap/affordable treatment	214	51.0
Good treatment experience	72	17.1
Ignorance	45	10.7
Fear of amputation	34	8.1
Uncertain	55	13.1

**Factors affecting willingness to patronize traditional bone setters in the future:** in Table 3, age, socio-economic status, having patronized traditional bone setters before, and being in support of surgeons and bone setters working together were significant predictors of willingness to patronize traditional bone setters in the future. The heads of households aged 30-39 years were twice less willing to patronize traditional bone setters compared to those 40 years and above (AOR=0.5, 95%CI: 0.2-0.9). Also, those in the low socio-economic class were twice as willing to patronize the traditional bone setters compared to the high socio-economic class (AOR=2.0, 95%CI: 1.2-3.3). The respondents who had patronized traditional bone setters before were 12 times more willing to patronize them in the future compared to their counterparts (AOR=12.5, 95%CI: 7.5-20.8). Those in support of surgeons and bone setters working together were about three times more willing to patronize the traditional bone setters compared to their counterparts (AOR=2.7, 95%CI: 1.6-4.4).

**Table 3:** factors affecting willingness to patronize traditional bone setters in the future among heads of households

Variable	Willing to patronize bone setters in the future (n=420)		X^2^ (p-value on bivariate analysis	AOR in multivariable analysis (95% CI)
	Yes N (%)	No N (%)		
**Age of respondents**				
<30 years	51 (30.7)	115 (69.3)	3.737(0.154)	0.6 (0.3 - 1.1)
30-39 years	39 (28.5)	98 (71.5)		0.5 (0.2 - 0.9)
≥40 years	46 (39.3)	71 (60.7)		1
**Gender**				
Male	74 (32.3)	155 (67.7)	0.001(0.975)	NA
Female	62 (32.5)	129 (67.5)		
**Marital status**				
Married	87 (33.9)	170 (66.1)	0.655(0.418)	NA
Single	49 (30.1)	114 (69.9)		
**Educational attainment of respondent**				
Tertiary education	83 (30.4)	190 (69.6)	1.394(0.238)	NA
Secondary education and below	53 (36.1)	94 (63.9)		
**Employment status of respondents**				
Unemployment	18 (38.3)	29 (61.7)	2.566(0.277)	NA
Self-employment	72 (34.4)	137(65.6)		
Salaried employment	46 (28.0)	118 (72.0)		
**Socio-economic status**				
Low socio-economic class	81 (38.2)	131 (61.8)	6.637(0.010)	2.0 (1.2 - 3.3)
High socio-economic class	55 (26.4)	153 (73.6)		1
**Have patronized bone setters before**				
Yes	95 (67.9)	45 (32.1)	120.708(<0.001)	12.5 (7.5 - 20.8)
No	41 (14.6)	239 (85.4)		1
**Supports surgeons and bone setters working together**				
Yes	76 (46.1)	89 (53.9)	23.227(<0.001)	2.7 (1.6 - 4.4)
No	60 (23.5)	195 (76.5)		1

## Discussion

The traditional bone setting is an unorthodox practice that has been in existence for a long time and is still being practiced despite decades of evolution in medicine including the availability of modern orthopaedic practice. In this study, the majority of the respondents 79.3%, have heard of traditional bone setters and their practices. This is similar to the findings of a study done in the same study area and outside Nigeria [4,13]. These studies reported that 84.5% and 95.1% of their respondents respectively had heard of traditional bone setters [4,13]. The probable explanation for the high level of awareness of the traditional bone setters is due to the increasingly uncontrolled promotion of information on unorthodox practices through the mass media on the ability of the traditional bone setters to cure all bone injuries [2]. About one-third of the respondents had patronized the traditional bone setters in the past and are willing to patronize them in the future. This contradicts the findings from studies in North-West and North-Central Nigeria which observed that a higher proportion of respondents patronized traditional bone setters [2,14]. However, our findings were similar to those of a study done in Southeastern Nigeria where this research was conducted [4]. This implies that in Nigeria, some people patronize traditional bone setters for the treatment of bone and joint injuries [1,4]. However, the higher prevalence noted in the other studies compared to our findings may be that patronizing traditional bone setters is linked with the cultural beliefs of the people [2,14].

The other researchers reported that the higher patronage of traditional bone setters in their study settings was because of the cultural beliefs of the people that the injury is based on supernatural influences that can be cured through unorthodox practices leading to more patronage of traditional bone setters [2,14]. This further explains our findings, where one-third of the respondents will encourage their family members to patronize traditional bone setters as against more than half of the respondents in North-Central Nigeria where the majority of their people patronize traditional bone setters [2]. Also, this study showed that the orthopaedic surgeons are perhaps better patronized by the respondents compared to traditional bone setters. This contrasted with other community-based studies done in North-Central and South-South Nigeria where the majority of their respondents preferred treatment of traditional bone setters [2,15]. The probable explanation for the differences in findings may be the type of respondents recruited in the studies. The subjects in this study were heads of households who are decision-makers in their various households and may be more knowledgeable on the type of health care to patronize [9].

The other studies recruited any member of the family and some of these participants may not have the appropriate information regarding the preferred treatment options as they may not be involved in family decision-making [2,15]. Despite the differences in the type of health personnel preferred, this study and other researchers in Africa [4,16] and beyond [17,18] noted that traditional bone setters have more treatment failures compared to orthopaedic surgeons. This may be linked to the fact that the traditional bone setters lack the basic medical training in modern orthopaedic care and infection prevention and control. This leads to their use of concoctions, unsanitary and tight splints on fractures resulting in compartment syndrome and gangrene [19].

It implies that the complications from traditional bone setters as a result of treatment failures increase the risk of mortality among people in the community. This necessitates collaboration between traditional bone setters and orthopaedic surgeons as suggested by the majority of the respondents in this study, by training the traditional bone setters not to exceed their area of competence and refer patients to orthopaedic surgeons before they develop complications [1].

The main reason that was given by the respondents, why people patronize the traditional bone setters in this study, was the affordability of treatment. This was consistent with other researchers´ findings in North-Central Nigeria, Banglore, and Turkey [2,18,20]. This shows that poverty plays a central role in the continued patronage of the traditional bone setters. The affordability of the treatment may be due to the use of herbal concoctions, wooden splints, and the application of special herbal balms which are cheaper compared to the expensive resources used in orthopaedic practice for care [21]. Also, traditional bone setters allow multiple little payments and payments in kind which prevents catastrophic health expenditure and hence promotes the patronage of traditional bone setters [2].

The study revealed that those aged 30-39 years were twice less willing to patronize traditional bone setters compared to those 40 years and above. Thus, those aged 40 years and above are more willing to patronize traditional bone setters and this corroborates with the findings of Aderibigbe *et al*. [2]. The possible explanation for this observation is that as individuals increase in age they tend to have more people who depend on them for financial support and this affects their health expenditures as they tend to reduce the cost of living [22]. In the older age group, it may lead to more willingness to patronize the traditional bone setters as the main reason given for patronizing the unorthodox practices in this study was the affordability of treatment.

Furthermore, those of low socio-economic class were about twice as willing to patronize the traditional bone setters compared to the high socio-economic class. This agrees with the research done in the same study setting and North-Central Nigeria [2,4]. It underscores the need to reduce the cost of health care in orthodox practices for better patronage as poverty plays a major role in the willingness to patronize traditional bone setters [21].

Despite the treatment failures associated with patronizing traditional bone setters that were noted in this study, surprisingly, those who had patronized traditional bone setters in the past were 12 times more willing to patronize them in the future when compared with those who have not utilized their services before. Also, the respondents who supported a collaboration between the two groups were about three times more willing to patronize the traditional bone setters compared to their counterparts. These may be linked to the treatment experiences of the individuals, in this study, some respondents reported that good treatment experience was one of the reasons people patronize traditional bone setters. Also, evidence has shown that the outcome of treatment by traditional bone setters is good for closed fractures of the shaft of the humerus, ulna, radius, and tibia but poor for peri-articular and open fractures [23]. However, there is still a need for collaboration between orthopaedic surgeons and traditional bone setters as was observed by respondents in this study. This is to ensure prompt referral of patients with musculoskeletal injuries that are beyond the competence of traditional bone setters to prevent treatment failures.

**Limitation of the study:** there could be the possibility of recall bias and social desirability bias among the respondents.

## Conclusion

The services of orthopaedic surgeons are preferable to the bone setters´, and both are also desired to work together. Cost and good treatment services play roles in the patronage of bone setters. Respondents who have patronized bone setters before are more willing to patronize them in the future. There is the need for bone setters to concentrate on areas of competence bearing in mind that there are measures of good in their services. Formal training of bone setters can help them enhance their skills and recognize when a referral is necessary.

### What is known about this topic

- Traditional bone setters are highly patronized in developing countries; they provide health care services that may result in acute or chronic complications due to poor medical skills.

### What this study adds

- This study accessed the heads of households who are decision-makers in the family and could influence the type of health care services their family members will utilize;
- Cost and good treatment services play roles in the patronage of bone setters.
